# Counting Limb Length Ratios in Roux-en-Y Gastric Bypass: A Demonstration of Safety and Feasibility Using a 25-Patient Case Series in a High-Volume Academic Center

**DOI:** 10.3390/jcm14155262

**Published:** 2025-07-25

**Authors:** Doua Elamin, Mélissa V. Wills, Juan Aulestia, Valentin Mocanu, Andrew Strong, Jerry Dang, Xiaoxi Feng, Matthew Kroh, Ricard Corcelles, Salvador Navarrete

**Affiliations:** Department of General Surgery, Cleveland Clinic, Cleveland, OH 44195, USA; elamind@ccf.org (D.E.); willsm@ccf.org (M.V.W.); aulestj@ccf.org (J.A.); mocanuv@ccf.org (V.M.); stronga3@ccf.org (A.S.); dangj3@ccf.org (J.D.); fengx4@ccf.org (X.F.); krohm@ccf.org (M.K.); corcelr@ccf.org (R.C.)

**Keywords:** Roux-en-Y gastric bypass, limb length, common channel, weight loss, obesity, bariatric surgery

## Abstract

**Background:** Despite being one of the most performed bariatric procedures, there is no consensus regarding optimal limb lengths for Roux-en-Y gastric bypass (RYGB), which may impact weight loss and obesity-related comorbidity resolution. We hypothesize that a ratio-adjusted small bowel to Roux and BP limb lengths in RYGB results in superior outcomes. **Objectives:** This study aims to define total intestinal length (TIL) and the feasibility of its intraoperative measurement during RYGB. The findings will serve as a foundation for a subsequent randomized trial evaluating different limb length ratios and their effect on postoperative outcomes. **Setting:** This was a single-center prospective cohort study conducted at Cleveland Clinic Foundation-Main Campus, a tertiary referral center in the United States. **Methods:** Between January and June 2023, 25 patients with BMI > 40 undergoing RYGB were enrolled. Total small bowel length was measured intraoperatively, and feasibility of measurement was assessed. Patient outcomes, including total weight loss, 30-day complications, and comorbidities at 1 year were captured. **Results:** Mean preoperative BMI was 47.6 ± 8.0 kg/m^2^. Mean total small bowel length was 592 ± 93.3 cm, with a mean biliopancreatic (BP) limb length of 109 ± 29 cm (18.86% ± 5.84 of total length) and Roux limb length of 103 ± 15 cm (17.71% ± 3.06 of total length). Measurement added an average of 11.5 min to operative time. Measurement feasibility was rated as “moderate” or easier in 80% of cases. One-year postoperative outcomes included a mean total weight loss of 31% and significant reductions in antihypertensive and anti-diabetic medication use. **Conclusions:** Total small bowel length measurement during RYGB is safe and feasible. High variability in bowel length was observed, with no significant correlation to demographic factors. Establishing individualized limb length ratios may improve weight loss outcomes and comorbidity resolution. Further studies are warranted to evaluate the impact of tailored limb length strategies.

## 1. Introduction

Obesity is a major health threat in the modern era, and the Roux-en-Y gastric bypass (RYGB) has been one of, if not the most important, bariatric procedures in the past five decades. Over this time, it has undergone multiple modifications of its technical aspects. There is tremendous heterogeneity in surgeons’ techniques, which often results in nuanced variations in the final RYGB anatomy. From surgeon to surgeon, there remain slight but possibly important differences in the size and shape of the pouch, anastomotic construction and configuration, and length of the different bowel limbs (Roux, biliopancreatic, and common channel) [1,2,3,4,5,6]. Understanding and tailoring these particular factors may herald a new era of patient-centered metabolic surgical care.

Currently there is no consensus on the lengths of the different limbs of the RYGB [7,8,9]. Most of the metabolic surgery community creates a Roux limb (RL) between 100 and 200 cm and a biliopancreatic (BP) limb between 50 and 150 cm, which may lead to a variability in outcomes in terms of weight loss and obesity-related comorbidity control [1,10,11]. Given the variability in bowel length between patients, the common channel length also differs and is not typically measured by most surgeons [9,12].

In the existing literature on small bowel limb lengths, there are several randomized controlled trials and comparative studies, but the results have not shown significant clinical outcome differences between different lengths in terms of weight loss and resolution of obesity-related comorbidities [7,13,14]. This may be due to inadequate methodological design, high variability in technical aspects, and differences in result reporting.

The current practice paradigm of most metabolic surgeons does not involve routine measurement of the entire length of small bowel during RYGB, and most employ absolute value limb lengths regardless of the total length of small bowel. We aim to ultimately investigate the impact of ratio-based limb lengths on clinical outcomes. To better understand the possible bowel length ratios, the first phase of our study consisted of measuring the bowel length in 25 patients who underwent RYGB. This will provide a better understanding of correlations and associations between post-RYGB intestinal anatomy and resultant weight loss and changes in metabolic syndrome among patients with obesity in our future study.

## 2. Methods

### 2.1. Study Design and Population

This was a single-center, preliminary prospective cohort study. The study aimed to examine the total small bowel length during planned RYGB to explore limb length ratios in our current practice and serve as a foundation for a larger prospective project comparing the effect of different limb lengths ratios during RYGB. Patients were recruited between January and June 2023, and all procedures were performed at Cleveland Clinic Foundation, Main Campus by 5 surgeons. Ethics approval was obtained from the Institutional Review Board (IRB #22-1405) and all patients provided informed consent.

Patients aged 18 to 70 years with obesity (BMI > 40) undergoing RYGB were included. Exclusion criteria were previous bariatric surgery, prior gastric or small bowel resections, and liver failure or transplantation. Patients underwent routine multidisciplinary preoperative evaluation, including assessments by Obesity Medicine, Nutrition, Psychiatry, and Bariatric Surgery teams.

### 2.2. Outcome Measures

The primary outcome was defining total intestinal length (TIL) in centimeters, the feasibility of measuring the intestinal lengths with grades of difficulty rated by the operating surgeon at the time of surgery based on a 5-point Likert scale, and time spent measuring the bowel.

Secondary outcomes included characterizing total weight loss (TWL) as measured by the difference in patient weight at various timepoints described below versus preoperative weight. Per the standard of care at our institution, routine postoperative follow-up in the multidisciplinary bariatric clinic occurred at 2 weeks, 1 month, 3 months, 6 months, and 12 months postoperatively. Assessment of postoperative TWL and BMI was evaluated at each interval time point. The presence of postoperative vitamin and nutritional deficiencies (namely iron, folic acid, vitamin B1, vitamin B12, vitamin D, calcium, zinc, copper, and albumin levels) was assessed at 6 and 12 months. Comorbidity data was collected, including pre- versus postoperative medication use, with attention to diabetes mellitus (DM), hypertension, hyperlipidemia, and Gastroesophageal Reflux Disease (GERD) medications. Thirty-day postoperative complications and adverse events were also tracked, as measured by the Clavien–Dindo Classification [15].

### 2.3. Surgical Procedure

All patients were placed in the supine position. Once pneumoperitoneum was created and the trocars were placed, the ligament of Treitz (LT) was identified. Small bowel length was measured with a laparoscopic instrument which was marked at 10 cm from the tip. To minimize technical bias, all participating surgeons reviewed a standardized instructional video on the bowel length measurement technique.

A dedicated continuous portion of the case was spent measuring the intestinal length, and this total time was recorded in minutes. The BP limb was defined as the length from the LT to the jejunojejunostomy (JJ). The RL was defined as the length from the gastrojejunostomy (GJ) to the JJ. The CC was defined as the length from the JJ to the terminal ileum. In this study, the roux and BP limbs were fashioned using pre-determined set lengths, as is the current standard of care at our institution. The ratio between the Roux limb and BP limb compared to TIL was then calculated. The gastric pouch was calibrated using a 40 Fr bougie. The GJ was performed using a 45 mm linear stapler introduced up to the 30 mm mark. The JJ was performed in a side-to-side fashion using a 60 mm tan load linear stapler. The GJ was assessed endoscopically at the end of the procedure. The mesenteric defects were closed using permanent suture. All patients were managed postoperatively with our standard ERAS protocol.

### 2.4. Statistical Analysis

This was a descriptive feasibility study designed to establish baseline parameters for a subsequent randomized trial. Continuous variables are presented as means ± standard deviations and categorical variables as frequencies and percentages. No formal hypothesis testing was performed given the exploratory nature and small sample size (n = 23) of this preliminary study.

## 3. Results

### 3.1. Clinical Demographics

Twenty-five (25) patients were enrolled from January to June 2023. Two patients were excluded due to significant pelvic adhesions that precluded safe bowel length measurement. The cohort included 23 females and 2 males with a mean age of 45.0 ± 10.3 years. The mean preoperative BMI was 47.5 ± 8.0 kg/m^2^. The comorbidities in this patient population were as follows: 11 patients had hypertension (44.0%), 9 patients had Type 2 DM (36.0%), 17 had Obstructive Sleep Apnea (OSA) (68.0%), 8 had GERD (32.0%), and 10 had hyperlipidemia (40.0%) (Table 1).

### 3.2. Perioperative Data

**Figure 1:**
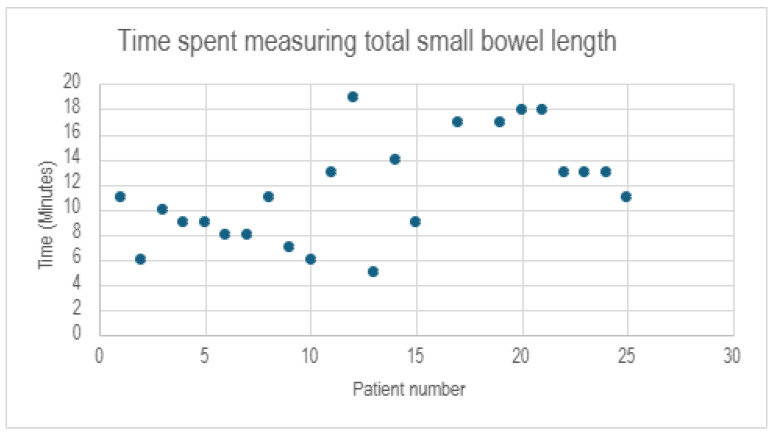
Time spent measuring bowel length.

All procedures were completed laparoscopically with an overall mean operative time of 142 min ± 28.6. There were no intraoperative complications, and no patients required blood products. The mean operative time dedicated to measuring the full length of the intestines was 11.52 min ± 4.19 (Figure 1).

One of our variables was measuring how difficult it was to count the length of the intestines. Surgeons were surveyed after each case and asked to rate the difficulty on a 5-point Likert scale. Two cases were not included due to adhesions requiring significant lysis with one of them undergoing sleeve gastrectomy instead of RYGB.

**Figure 2:**
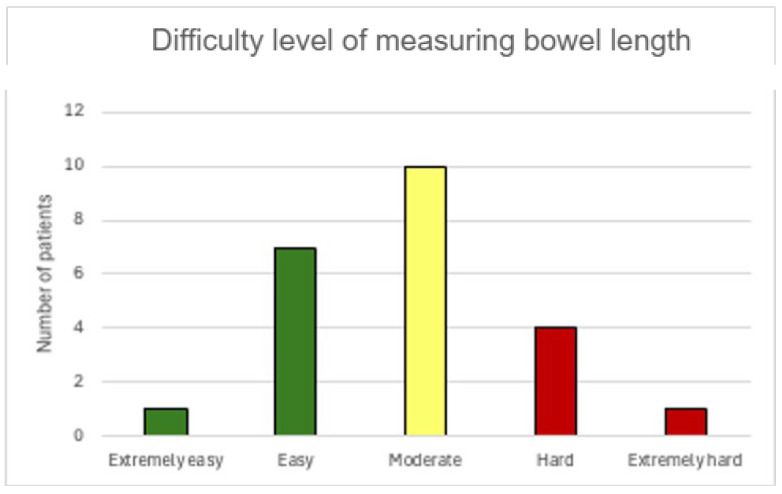
Difficulty levels when measuring bowel length.

Of the 23 cases included, 1 case was rated “Extremely easy” (4.3%), 7 cases were rated “Easy” (30.4%), 10 cases were rated “Moderate” (43.5%), 4 were “Hard” (17.4%), and 1 case “Extremely Hard” (4.3%) which indicates that it is feasible to count small bowel length without major difficulties in about 80% of the patients (Figure 2). In the remaining 20% (hard to extremely hard cases), the reason was mainly attributed to the amount of mesenteric and visceral fat encountered.

**Figure 3:**
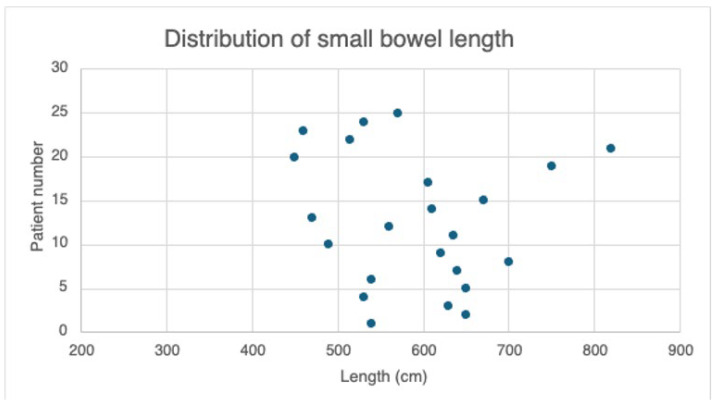
Distribution of total small bowel length.

The mean total limb length was 592 cm ± 93.34, with a minimum bowel length of 450 cm and a maximum of 820 cm (Figure 3). The mean BP limb length was 109 cm ± 29, while the mean ratio was 18.86% ± 5.84. The mean Roux limb length was 103 cm ± 15, and the mean ratio was 17.71% ± 3.06. The mean common channel length was 379 cm ± 90, and the mean ratio was 63% ± 6.78.

The mean preoperative weight was 126 kg (84.8–160.4). The mean preoperative BMI was 47.6 (40–66.9). The mean 1-year postoperative weight was 87 kg (65–118) and mean BMI was 32.9 (23–43). The mean 1-year TWL was 31%.

### 3.3. Thirty-Day Postoperative Complications

In our cohort, two patients had prolonged hospital stays: one required an additional 6 days due to a Clostridium difficile infection, while another was hospitalized for 5 days due to intractable nausea. One patient required two emergency department visits for dehydration.

### 3.4. Comorbidities and Outcomes at 1 Year

All patients had follow-up at twelve months. Of the 44% of patients with HTN, 36% (four patients) were using more than one anti-hypertensive medication. One year postoperatively, 91% had a decrease in medications used, and 55% were not on any anti-hypertensive medications. Three of nine patients with Type 2 DM were using more than one anti-glycemic agent in the form of oral medications or long-acting and/or short-acting insulin. A total of 78% were using fewer medications at one year, and 71% of those were completely off anti-glycemic medications. One patient was on two oral medications and short-acting insulin preoperatively and was able to successfully control their glucose levels postoperatively without any medications. A total of 69% of patients using CPAP were off CPAP one year postoperatively (Table 2). Postoperative nutritional information and vitamin levels at 6–12 months (±3 months) are included in Appendix A (Table A1).

## 4. Discussion

The available literature regarding the effect of different ratios between RL/BP and CC length is scant. Common channel length is reported to be variable between individuals [16,17]. Studies examining weight loss after duodenal switches have affirmed the significant role reduced common channel lengths play in weight loss, often at the price of nutritional deficiencies and diarrhea [18]. The optimal ratio between the limb lengths to guarantee the best weight loss and disease control with the lowest morbidity profile is still not known, limiting our ability to deliver personalized metabolic procedures.

This preliminary study marks the initial phase of a broader trial exploring the impact of biliopancreatic limb lengthening on weight loss and comorbidity management following RYGB. In this initial phase, we measured the total small intestine length and calculated BP limb ratios. Our subsequent phase will randomize patients into groups with different limb length ratios.

To establish a control group for comparison with the intervention arm, we first assessed the current surgical practices at our institution by analyzing both absolute numbers and corresponding ratios. This involved measuring total bowel length and determining the average ratio currently used in RYGB procedures performed by our surgeons.

We found that measuring the entire intestinal length is feasible and does not significantly prolong operating time. This process was manageable for all surgeons in the group in about 80% of cases. There was no modification in the technique needed, like placing another trocar or changing trocar positions. With simple modifications of patient positioning, we were able to measure the bowel length without difficulty. To our knowledge, this is the first paper addressing the difficulty of measuring the bowel in laparoscopic bariatric surgery.

Operative time is one of the limitations of measuring small bowel length in surgery described in the literature. Isreb et al. studied the effect of marked instruments versus surgeons’ estimation of bowel length [19]. They concluded that time was almost doubled using marked instruments but provided a statistically significant greater accuracy of bowel length regardless of surgeon experience. The multicenter Dutch Common Channel Trial (DUCATI) compared standard vs. distal RYGB with similar biliopancreatic bowel limb lengths [5]. The total small bowel length was measured at each procedure, and a 7 min difference was quoted as acceptable increase in surgical time for adequate small bowel measurement when comparing their total procedure time to RYGB performed in another center not participating in the study. This study, however, did not record the duration of time spent in each case for measuring small bowel length but merely compared the differences in average total surgery duration. Another feared complication is small bowel injury during laparoscopic surgery. The incidence of injury is small and is usually discovered intraoperatively or within 24 h thereafter [20]. We did not incur bowel injury in our cohort.

Hess et al. reported on the outcomes of open biliopancreatic diversion with duodenal switch via midline laparotomy, detailing a methodology in which the entire length of the small intestine is measured from the cecum to the ligament of Treitz, with surgical limbs constructed as a percentage of this measurement [21]. Despite our smaller sample size, their study aligns with our findings in demonstrating variability in bowel lengths among patients, with a minimum TIL of 473 cm compared to 450 cm in our cohort. The maximum TIL in their population was 1065 cm, with a mean length of 722 cm. In contrast, our study reported a maximum TIL of 820 cm, with a mean of 590 cm and a standard deviation of 90 cm. Notably, Hess et al. did not provide information regarding the distribution of TIL in their sample [21]. Although this paper was published in 1998, it remains a significant reference, highlighting that intestinal length variability is an important factor to consider intraoperatively.

A study by Tachinno also found similar intestinal lengths and high variability in laparotomy patients, with comparable results in laparoscopy, though laparoscopic data was not included [22]. The study also identified correlations between height, sex, and total intestinal length, similarly to a study by Liu et al. proving a positive correlation between intestinal length and anthropometrics [23]. However, in our study, there was no correlation between intestinal length, age, sex, BMI, or height, which could be related to our small sample.

Interestingly, our average ratio for the length of the Roux limb and BP limb was 17%, which corresponded to 100 cm. This allowed us to have a reference for the control group in the second phase of the trial. The weight loss observed in the first year of the RYGB was as expected, with an average of 31% of TWL. There was no correlation between the total intestinal length and weight loss and comorbidity control. These findings are similar to the ones described by Tachinno and Abellan [22,24].

Measuring the whole intestinal length in the bariatric surgery community is not popular. There is the perception that measuring represents a technical challenge [25,26]. However, we believe that every patient who undergoes a bariatric procedure where there is intestinal involvement should have the total intestinal length counted. In our study, we found that 16% (four of our patients) had intestines shorter than 500 cm long, and 12% (3 patients) had a TIL longer than 680 cm. This suggests that we could be under- or overtreating 26% of our patients, emphasizing the importance of measuring the bowel.

The primary limitation of our study is the small sample size, which limited the statistical power to identify potential correlations between weight loss and variations in intestinal length. However, it is important to note that this was not the aim of the study. Our cohort was relatively homogenous, with the majority of patients being female (92%), and it was a single-institution study. Both of these factors may limit generalizability.

Although our practice guideline is to measure vitamin and micronutrient levels at 6- and 12-month postoperative intervals, this did not occur precisely at that time period for most of our patients. We suspect that the variable time points within which these levels were obtained depend on patient-specific needs and visit timings. As a result, there was inconsistency in the availability and completeness of nutritional data at the predefined 6- and 12-month marks across the cohort, limiting interpretability.

Another notable challenge was the reluctance of some surgeons to measure intestinal length, attributing this to technical difficulties, which may have introduced variability in data collection, and although the measuring technique was standardized, we cannot account for possible measurement errors. Furthermore, although all participating surgeons received standardized instructions via a training video and were briefed on a uniform measurement protocol, subtle differences in surgical technique, experience, and perception of feasibility may still have influenced outcomes. Importantly, all instruments used to measure the bowel were marked in the same way. The measurement variability could also be attributed to the degree of bowel tension (i.e., over- or under-stretching) during measurement. We believe this was minimized following the instructional video and protocol briefing, though we acknowledge that without direct validation like repeat measurements or inter-observer checks, residual variability cannot be ruled out.

While five surgeons participated in this study, we did not perform a formal analysis stratified by individual surgeons. We recognize this as a limitation and believe that future studies should include surgeon-stratified analyses to better assess reproducibility and technique-related variability in bowel measurement.

Additionally, the sample size was insufficient to encompass the full spectrum of intestinal length variability across the broader population [27], and our follow-up was limited to one year postoperatively, which may not address the durability of weight loss or late effects of limb lengths. Technical challenges were also encountered during intestinal measurements, particularly in patients with a history of previous pelvic surgeries or those with a heavier omentum, which further complicated accurate assessments.

## 5. Conclusions

This study demonstrates that intraoperative measurement of total intestinal length during RYGB is both safe and feasible, adding only 11.5 min on average to operative time. We observed high variability in intestinal length, with no correlation of intestinal length to the demographic variables. We obtained the average ratio of intestinal limb length, which will be used as a control group in the second phase of the study.

Our findings suggest that routine measurement of intestinal length may be beneficial to better tailor metabolic operations to the individual patient. Long-term prospective studies are needed to evaluate outcomes with different limb length ratios.

## Figures and Tables

**Table 1:** Demographics and baseline characteristics of patients.

Characteristic	Count (N) or Mean (±SD)
Age at time of surgery (years)	45.0 ± 10.3
Female	23 (92.0%)
Race	
White	13 (52.0)
Unknown/not reported	6 (24.0)
Black	3 (12.0)
Other	3 (12.0)
Mean preoperative BMI (kg/m^2^) ± SD	47.6 ± 8.0
Hypertension	11 (44.0)
Taking ≥1 antihypertensives	11 (44.0)
Diabetes	
Non-diabetic or diet-controlled	16 (64.0)
Type I diabetes mellitus	0 (0.0)
Type II diabetes mellitus	9 (36.0)
On short- or long-acting insulin	7 (28.0)
Taking non-insulin medication	7 (28.0)
Obstructive sleep apnea	17 (68.0)
Gastroesophageal reflux disease	8 (32.0)
Hyperlipidemia	10 (40.0)

Abbreviations: standard deviation (SD); body mass index (BMI).

**Table 2:** Changes in postoperative medication use.

	Medications Used Preop	Decrease in Medications Postop n (%)	Cessation of Medications Postop n (%)
HTN	11	10 (91)	6 (55)
Type 2 DM	9	7 (78)	5 (56)

## Data Availability

The original contributions presented in this study are included in the article. Further inquiries can be directed to the corresponding author(s).

## Appendix A

**Table A1:** Nutritional and Vitamin levels at 6 and 12 months (+/−3 months) postoperatively.

Patient ID	Iron 6 mo	Iron 1 yr	Folate 6 mo	Folate 1 yr	Vitamin B1 6 mo	Vitamin B1 1 yr	Vitamin B12 6 mo	Vitamin B12 1 yr	Vitamin D 6 mo	Vitamin D 1 yr	Calcium 6 mo	Calcium 1 yr	Zinc 6 mo	Zinc 1 yr	Copper 6 mo	Copper 1 yr	Albumin 6 mo	Albumin 1 yr
1	89	94	20	17	155.4	-	844	691	28.8	27.5	9.6	9.7	-	76	-	101	4.3	4.3
2	-	-	-	-	-	-	-	-	-	-	-	-	-	-	-	-	-	-
3	83	91	-	8.9	201	183.5	970	595	26.2	22	10.1	9.6	-	57	-	86	4.8	4.4
4	-	-	-	-	-	-	-	-	-	-	8.9	9.4	-	67	-	85	4.3	4.6
5	107	-	6.9	-	113.8	-	182	-	21.2	-	9.9	-	63	-	94	-	4.6	-
6	66	76	>20	>20	-	198.1	586	883	44.2	57.1	9.3	9	-	84	-	134	3.9	4.3
7	-	-	-	-	-	-	-	-	-	-	-	-	-	-	-	-	-	-
8	-	-	-	-	-	-	-	-	-	-	-	-	-	-	-	-	4.1	4
9	-	-	-	-	-	-	-	-	-	-	-	-	-	-	-	-	-	-
10	-	-	-	-	-	-	-	-	-	-	-	-	-	-	-	-	-	-
11	-	-	-	-	-	-	-	-	-	-	9.6	-	-	-	-	-	4.6	-
12	-	-	-	-	-	-	-	-	-	-	9.7	-	-	-	-	-	-	-
13	-	69	-	18.9	-	226.5	-	1100	-	42.2	-	9.7	-	54	-	117	-	4.3
14	-	-	-	-	-	-	-	-	-	-	-	9.2	-	-	-	-	-	4.3
15	-	-	-	-	-	-	-	-	-	-	-	-	-	-	-	-	-	-
16	-	-	-	-	-	-	-	-	-	-	-	-	-	-	-	-	-	-
17	-	-	-	6.8	-	-	-	263	-	-	-	9.1	-	-	-	-	-	4.2
18	90	-	17.5	-	178.1	-	376	-	49.3	-	10.3	9.9	69	-	112	-	3.7	3.8
19	140	-	11.7	-	-	-	367	-	23.9	-	9.5	8.9	-	-	-	-	3.9	3.9
20	-	-	-	-	-	-	-	-	-	-	-	-	-	-	-	-	-	-
21	37	-	-	-	80.2	-	776	-	30.7	-	9.5	-	57	-	78	-	3.8	-
22	-	-	-	-	-	-	-	250	-	33.3	-	8.6	-	-	-	-	-	-
23	-	-	-	-	-	-	-	-	-	-	9.2	-	-	-	-	-	-	-
24	-	-	-	-	-	-	-	-	-	-	-	8.8	-	-	-	-	4	4.3
25	-	-	-	-	-	-	-	-	-	-	-	-	-	-	-	-	-	-

## References

[1] Eagleston J., Nimeri A. (2023). Optimal Small Bowel Limb Lengths of Roux-en-Y Gastric Bypass. Curr. Obes. Rep..

[2] Haghighat N., Kamran H., Moaddeli M.N., Hosseini B., Karimi A., Hesameddini I., Amini M., Hosseini S.V., Vahidi A., Moeinvaziri N. (2022). The impact of gastric pouch size, based on the number of staplers, on the short-term weight outcomes of Roux-en-Y gastric bypass. Ann. Med. Surg..

[3] Stumpf O., Lange V., Rosenthal A., Lefering R., Paasch C. (2022). A 30 mm sized gastrojejunostomy may lead to a lower rate of therapy failure in comparison to a 45 mm sized gastrojejunostomy following laparoscopic Roux-en-Y gastric bypass. Ann. Med. Surg..

[4] Kamocka A., Chidambaram S., Erridge S., Vithlani G., Miras A.D., Purkayastha S. (2022). Length of biliopancreatic limb in Roux-en-Y gastric bypass and its impact on post-operative outcomes in metabolic and obesity surgery-systematic review and meta-analysis. Int. J. Obes..

[5] Gadiot R.P., Leeman M., Biter L.U., Dunkelgrun M., Apers J.A., Hof G.V., Feskens P.B., Mannaerts G.H. (2020). Does the Length of the Common Channel as Part of the Total Alimentary Tract Matter? One Year Results from the Multicenter Dutch Common Channel Trial (DUCATI) Comparing Standard Versus Distal Roux-en-Y Gastric Bypass with Similar Biliopancreatic Bowel Limb Lengths. Obes. Surg..

[6] Boerboom A., Cooiman M., Aarts E., Aufenacker T., Hazebroek E., Berends F. (2020). An Extended Pouch in a Roux-En-Y Gastric Bypass Reduces Weight Regain: 3-Year Results of a Randomized Controlled Trial. Obes. Surg..

[7] Hort A., Cheng Q., Morosin T., Yoon P., Talbot M. (2023). Optimal common limb length in Roux-en-Y gastric bypass surgery: Is it important for an ideal outcome?—A systematic review. ANZ J. Surg..

[8] Dang J.T., Deprato A., Verhoeff K., Sun W., Pandey A., Mocanu V., Karmali S., Switzer N.J., Nguyen N.T. (2022). Variation of Laparoscopic Roux-en-Y Gastric Bypass Techniques: A Survey of 518 Bariatric Surgeons. Obes. Surg..

[9] Kumar P., Yau H.C.V., Trivedi A., Yong D., Mahawar K. (2020). Global Variations in Practices Concerning Roux-en-Y Gastric Bypass-an Online Survey of 651 Bariatric and Metabolic Surgeons with Cumulative Experience of 158,335 Procedures. Obes. Surg..

[10] Estrada A., Rodriguez-Quintero J.H., Pereira X., Moran-Atkin E., Choi J., Camacho D. (2024). Gastric bypass revisional surgery: Percentage total body weight loss differences among three different techniques. Langenbecks Arch. Surg..

[11] Süsstrunk J., Lazaridis I.I., Köstler T., Kraljević M., Delko T., Zingg U. (2021). Long-Term Outcome of Proximal Versus Very-Very Long Limb Roux-en-Y Gastric Bypass: The Roux-Limb to Common Channel Ratio Determines the Long-Term Weight Loss. Obes. Surg..

[12] Wang A., Poliakin L., Sundaresan N., Vijayanagar V., Abdurakhmanov A., Thompson K.J., Mckillop I.H., Barbat S., Bauman R., Gersin K.S. (2022). The role of total alimentary limb length in Roux-en-Y gastric bypass: A systematic review. Surg. Obes. Relat. Dis. Off. J. Am. Soc. Bariatr. Surg..

[13] Stevenson M., Lau R., Brathwaite C.E.M., Ragolia L. (2024). Beyond Measure: Navigating the Complexities of Limb Length Optimization in Roux-en-Y Gastric Bypass Surgery. Obes. Surg..

[14] Eckharter C., Heeren N., Mongelli F., Sykora M., Fenner H., Scheiwiller A., Metzger J., Gass J.M. (2022). Effects of short or long biliopancreatic limb length after laparoscopic Roux-en-Y gastric bypass surgery for obesity: A propensity score-matched analysis. Langenbecks Arch. Surg..

[15] Clavien P.A., Barkun J., de Oliveira M.L., Vauthey J.N., Dindo D., Schulick R.D., de Santibañes E., Pekolj J., Slankamenac K., Bassi C. (2009). The Clavien-Dindo classification of surgical complications: Five-year experience. Ann. Surg..

[16] Kassir R., Blanc P., Vola M., Tiffet O. (2016). Common Limb Length Does Not Influence Weight Loss After Standard Laparoscopic Roux-En-Y Gastric Bypass. Obes. Surg..

[17] Mahawar K.K., Kumar P., Parmar C., Graham Y., Carr W.R., Jennings N., Schroeder N., Balupuri S., Small P.K. (2016). Small Bowel Limb Lengths and Roux-en-Y Gastric Bypass: A Systematic Review. Obes. Surg..

[18] Leeman M., Gadiot R.P., Wijnand J.M., Birnie E., Apers J.A., Biter L.U., Dunkelgrun M. (2020). Effects of standard v. very long Roux limb Roux-en-Y gastric bypass on nutrient status: A 1-year follow-up report from the Dutch Common Channel Trial (DUCATI) Study. Br. J. Nutr..

[19] Isreb S., Hildreth A.J., Mahawar K., Balupuri S., Small P. (2012). Laparoscopic Instruments Marking Improve Length Measurement Precision. World J. Laparosc. Surg..

[20] van der Voort M., Heijnsdijk E.A.M., Gouma D.J. (2004). Bowel injury as a complication of laparoscopy. Br. J. Surg..

[21] Hess D.S., Hess D.W. (1998). Biliopancreatic diversion with a duodenal switch. Obes. Surg..

[22] Tacchino R.M. (2015). Bowel length: Measurement, predictors, and impact on bariatric and metabolic surgery. Surg. Obes. Relat. Dis. Off. J. Am. Soc. Bariatr. Surg..

[23] Liu Z., Huang Z., Zhang Y., Gao L., Yang Y., Chen X., Zhao W., Ma L., Wang Y., Dong Z. (2024). Correlation of T2DM and Anthropometric Measures with Total Small Bowel Length and Its Effects on Diabetes Remission After Bariatric Surgery. Obes. Surg..

[24] Abellan I., Luján J., Frutos M.D., Abrisqueta J., Hernández Q., López V., Parrilla P. (2014). The influence of the percentage of the common limb in weight loss and nutritional alterations after laparoscopic gastric bypass. Surg. Obes. Relat. Dis. Off. J. Am. Soc. Bariatr. Surg..

[25] Slagter N., van Wilsum M., de Heide L.J., Jutte E.H., Kaijser M.A., Damen S.L., van Beek A.P., Emous M. (2022). Laparoscopic Small Bowel Length Measurement in Bariatric Surgery Using a Hand-Over-Hand Technique with Marked Graspers: An Ex Vivo Experiment. Obes. Surg..

[26] Käkelä P., Rantanen T., Virtanen K.A. (2021). The Importance of Intestinal Length in Triglyceride Metabolism and in Predicting the Outcomes of Comorbidities in Laparoscopic Roux-en-Y Gastric Bypass—A Narrative Review. Obes. Surg..

[27] Almalki O.M., Soong T.C., Lee W.J., Chen J.C., Wu C.C., Lee Y.C. (2021). Variation in Small Bowel Length and Its Influence on the Outcomes of Sleeve Gastrectomy. Obes. Surg..

